# MicroRNA-421 regulated by HIF-1α promotes metastasis, inhibits apoptosis, and induces cisplatin resistance by targeting E-cadherin and caspase-3 in gastric cancer

**DOI:** 10.18632/oncotarget.8228

**Published:** 2016-03-21

**Authors:** Xiaoxiao Ge, Xinyang Liu, Fengjuan Lin, Peng Li, Kaiyi Liu, Ruixuan Geng, Congqi Dai, Ying Lin, Wenbo Tang, Zheng Wu, Jinjia Chang, Jianwei Lu, Jin Li

**Affiliations:** ^1^ Department of Medical Oncology, Fudan University, Shanghai Cancer Center, Shanghai, 200000, P.R. China; ^2^ Department of Oncology, Shanghai Medical College, Fudan University, Shanghai, 200000, P.R. China; ^3^ Shanghai Medical College, Fudan University, Shanghai, 200000, P.R. China; ^4^ Harvard T.H. Chan School of Public Health, Boston, MA, 02115, U.S.A; ^5^ Department of Regenerative Medicine and Stem Cell Research Center, Tongji University School of Medicine, Shanghai, 20000, P.R. China; ^6^ International Medical Services, Peking Union Medical College Hospital, Beijing, 100000, P.R. China; ^7^ The Advanced Institute of Translational Medicine and School of Software Engineering, Tongji University, Shanghai, 200073, P.R. China

**Keywords:** gastric cancer, miR-421, HIF-1α, cisplatin resistance, epithelial-mesenchymal transition

## Abstract

Hypoxia and dysregulation of microRNAs (miRNAs) have been identified as crucial factors in carcinogenesis. However, the potential mechanisms of HIF-1α and miR-421 in gastric cancer have not been well elucidated. In this study, we found that miR-421 was up-regulated by HIF-1α. Overexpression of miR-421 promoted metastasis, inhibited apoptosis, and induced cisplatin resistance in gastric cancer *in vivo* and *in vitro*. E-cadherin and caspase-3 were identified as targets of miR-421. Besides, relative mRNA expression of miR-421 was significantly increased in gastric cancer tumor tissues compared with non-tumor tissues in a cohort of gastric cancer specimens (n=107). The expression of miR-421 was higher in advanced gastric cancers compared with localized ones. Moreover, Kaplan–Meier analysis illustrated that those patients with low levels of miR-421 had a significant longer overall survival (p = 0.006) and time to relapse (p = 0.007). Therefore, miR-421 could serve as an important prognostic marker and a potential molecular target for therapy in gastric cancer.

## INTRODUCTION

Hypoxia, a common feature of rapidly growing tumors, is associated with various physiological and pathophysiological events, including carcinogenesis, cancer cell migration and invasion, angiogenesis and chemotherapy resistance [1, 2]. These changes are regulated by a series of hypoxia-induced genes [3]. HIF-1α, induced by hypoxia, is regarded as the key transcription factor in downstream regulation [3], and activates a signaling transduction network including cell cycle, cell apoptosis and metastasis which drives the adaptation of cancer cells under hypoxic conditions to a more aggressive phenotype [4]. Previous studies illustrated the correlation between drug resistance and epithelial-mesenchymal transition (EMT), and indicated that EMT played a critical role in chemotherapy resistance [5–7]. Moreover, inhibition of cell apoptosis was another important process in decreasing the chemotherapy sensitivity [8]. However, the correlation among HIF-1α, EMT, and apoptosis and its underlying mechanisms in regulation of chemotherapy resistance have not been well elucidated.

microRNAs (miRNAs) are evolutionally conserved, endogenous, non-coding, small (20~23 nucleotides) RNAs, which regulate gene expression at post transcriptional level [9]. miRNAs accelerate the degradation and/or block the translation of their target mRNAs to induce post-transcriptional gene repression, and regulate various cellular processes such as proliferation, differentiation, metabolism and apoptosis [10]. Loss expression of miRNA genes was firstly observed in leukemia, and demonstrated the critical role of miRNAs in carcinogenesis [11]. Subsequent researches illustrated the carcinogenesis of miRNAs in many types of cancer [7, 12–14].

Recent researches illustrated that hypoxia regulated a series of miRNAs, which coordinated with HIF-1α to modulate cell proliferation, metastasis, apoptosis and angiogenesis [8, 15–17]. miR-210, a classic HIF-1α induced miRNA, has been reported to be involved in many aspects of carcinogenesis [18]. HIF-1α induced miR-382 has also been reported as an angiogenic miRNA by suppressing the expression of PTEN [15]. Recently, miR-421 has been found to be able to promote cell proliferation and migration in hepatocellular carcinoma, nasopharyngeal carcinoma, and neuroblastoma [19–21]. However, little is known about its role under hypoxia in gastric cancer.

Therefore, in our study, we investigated the roles of HIF-1α induced miR-421 in gastric cancer, and we found that miR-421 promoted metastasis, inhibited apoptosis, and induced cisplatin resistance by targeting E-cadherin and caspase-3.

## RESULTS

### Elevated HIF-1α induces cisplatin resistance in gastric cancer

**Figure 1:**
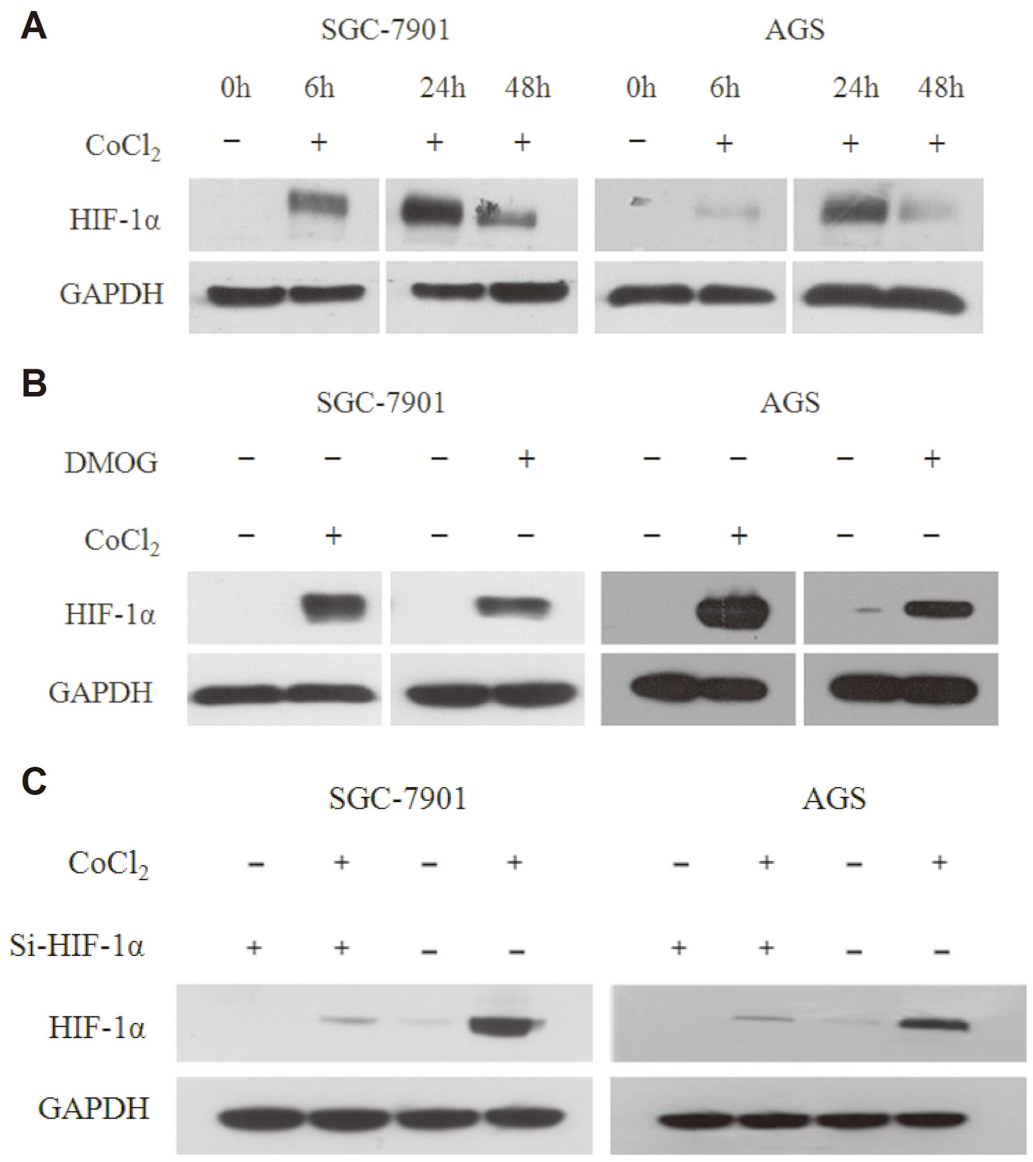
HIF-1α expression is stabilized by CoCl2 and DMOG **A.** The expression of HIF-1α increases in SGC-7901 and AGS gastric cancer cell lines, which were treated with the hypoxia mimetic agent CoCl_2_ (100μM) from 0h to 48h, and reaches the highest point at 24h. **B.** Western blot analysis indicates that CoCl_2_ makes the expression of HIF-1α more stable in gastric cell lines compared to DMOG. Both cell lines were treated with CoCl_2_ and DMOG (300μM) for 24h. **C.** Western blot analysis indicates that co-transfected Si-HIF-1α (50μM) with CoCl2 could reverse the stabilized effect of CoCl_2_ on HIF-1α in both cell lines.

In order to verify the change of HIF-1α under hypoxia in gastric cell lines, we used cohalt chloride (CoCl_2_) and Dimethyloxalylglycine (DMOG) to simulate the hypoxia environment. The SGC-7901 and AGS gastric cancer cell lines were treated from 0h to 48h with the hypoxia mimetic agent CoCl_2_ (100μM). The expression of HIF-1α increased in both cell lines and reached the highest point at 24h (Figure 1A). Next, we used CoCl_2_ and DMOG (300μM) to treat both cell lines for 24h. The result indicated that CoCl_2_ made the protein level of HIF-1α more stable in gastric cell lines compared to DMOG (Figure 1B). To further testify the function of HIF-1α, we co-transfected Si-HIF-1α (50μM) with CoCl_2_ to detect the expression of HIF-1α, and we found that Si-HIF-1α could reverse the stabilized effect of CoCl_2_ on HIF-1α in both cell lines (Figure 1C). Therefore, we chose CoCl_2_ for the follow-up experiments.

**Figure 2:**
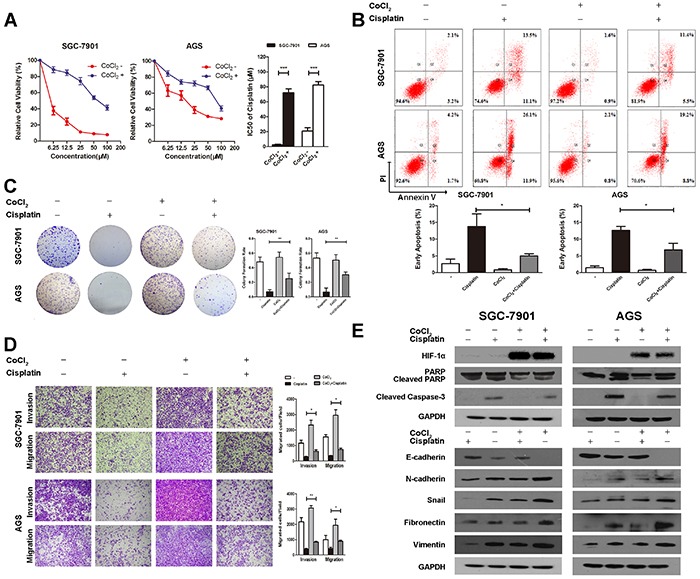
Elevated HIF-1α induces cisplatin resistance in gastric cancer **A.** The IC50 of cisplatin in SGC-7901 and AGS is 2.4 ± 0.66 μM and 20.8 ± 4.50 μM respectively. After being treated with CoCl_2_ for 24h, the IC50 of cisplatin in SGC-7901 increases to 72.1 ± 5.20 μM, and AGS to 82.3 ± 4.70 μM. Error bars mean 95 % confidence intervals. Columns mean of three independent experiments, and bars SD. **B.** Flow cytometry analysis indicates that cisplatin drastically increases the percentage of early apoptotic cells in both cell lines, which could be attenuated by induction of CoCl_2_. Representative images and quantitative analysis of cell apoptosis are presented. Columns mean of three independent experiments, and bars SD. **C.** CoCl_2_ increases the colony forming ability in cisplatin treated gastric cell lines compared with those treated with cisplatin alone. Representative images and quantitative analysis of colony formation rate are presented. Columns mean of three independent experiments, and bars SD. **D.** Transwell assays illustrate that cisplatin decreases the migration and invasion ability of both cell lines, and the inhibitory effect could be reversed by induction of CoCl_2_ Representative migration and invasion images at ×100, and quantitative analysis of transwell assays are presented. Columns mean of three independent experiments, and bars SD. **E.** Western blot analysis shows that the expression of HIF-1α is significantly increased by CoCl_2_. The expression of cleaved caspase-3 and cleaved PARP are induced by cisplatin, E-cadherin is increased, and N-cadherin, Fibronectin, Vimentin, and Snail are decreased by cisplatin, which could be reversed by CoCl_2_ in both cell lines. The E-cadherin is significantly down regulated, whereas N-cadherin, Fibronectin, Vimentin, and Snail are up regulated when treated with CoCl_2_ alone. *P<0.05; **P<0.01; ***P < 0.001.

To explore the biological function of HIF-1α in gastric cancer, cisplatin-induced anti-cancer function was analyzed in SGC-7901 and AGS gastric cancer cell lines under hypoxia. The IC50 of cisplatin in SGC-7901 and AGS gastric cancer cell lines were 2.4 ± 0.66 μM and 20.8 ± 4.50 μM. After treated with CoCl_2_ for 24h, the IC50 of cisplatin in both cell lines increased to 72.1 ± 5.20 μM and 82.3 ± 4.70μM, respectively (Figure 2A).

Flow cytometry was conducted to determine whether cisplatin induced apoptosis was affected by hypoxia. The gastric cell lines treated with DMSO, the solvent of cisplatin, were used as controls. Flow cytometry analysis indicated that cisplatin drastically increased the percentage of early apoptotic cells in both cell lines. The apoptotic ability was attenuated by induction of CoCl_2_ (Figure 2B). Furthermore, CoCl_2_ increased the colony forming ability in cisplatin treated gastric cell lines. Compared with cell lines treated cisplatin alone, the colony formation ability increased drastically in cisplatin and CoCl_2_ group (Figure 2C). Besides, western blotting analysis indicated that cisplatin significantly elevated the expression of cleaved caspase-3 and cleaved PARP, which could be reversed by induction of CoCl_2_, indicating that CoCl_2_-induced HIF-1α suppressed the cisplatin-induced apoptosis (Figure 2E).

To further elucidate the function of HIF-1α in gastric cancer, we investigated the effect of CoCl_2_ on cisplatin-treated SGC-7901 and AGS cell motility via transwell assay. The results suggested that cisplatin decreased the migration and invasion ability of both cell lines, and the inhibitory effect could be reversed by induction of CoCl_2_ (Figure 2D). EMT is an important process in invasion and metastasis of cancer and the loss expression of E-cadherin is a key factor in EMT. Western blotting was used to analyze the expression of EMT-related proteins in cisplatin-treated cell lines treated with CoCl_2_. As a result, the expression of E-cadherin was increased, and that of N-cadherin, Fibronectin, Vimentin and Snail was decreased by cisplatin. However, the increased expression of E-cadherin and the decreased expression of N-cadherin, Fibronectin, Vimentin and Snail were attenuated by induction of CoCl_2_ in both cell lines (Figure 2E). Besides, we also found that the epithelial biomarker E-cadherin was significantly down regulated, whereas the mesenchymal biomarkers N-cadherin, Fibronectin, Vimentin and Snail were up regulated when treated with CoCl_2_ alone (Figure 2E).

### HIF-1α is involved in miR-421 expression

**Figure 3:**
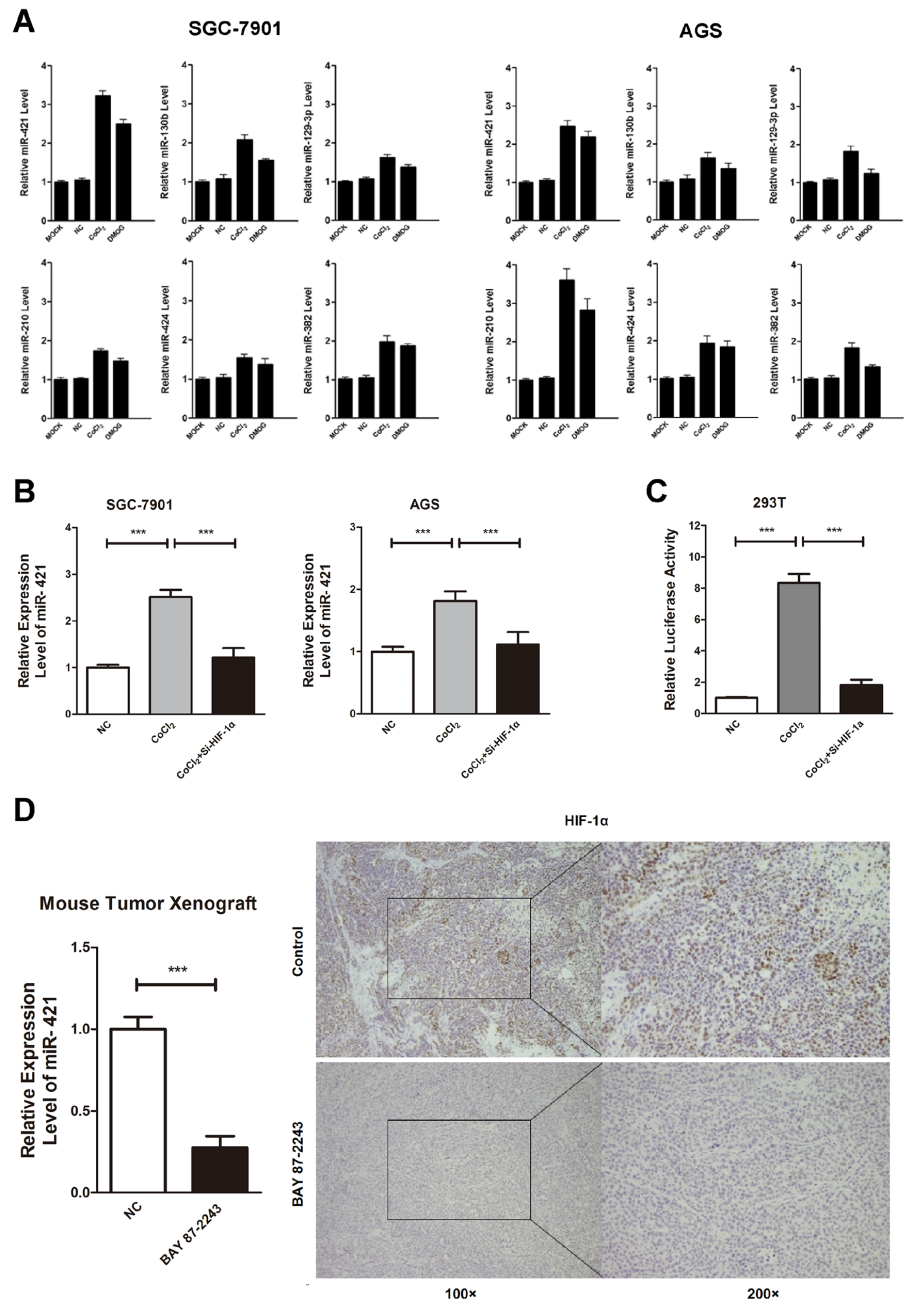
HIF-1α is involved in miR-421 expression **A.** Quantitative real-time PCR (qRT-PCR) was conducted to confirm the microchip data, and miR-421, miR-130b, miR-129-3p, miR-210, miR-424 and miR-382 were drastically up regulated after being treated with CoCl_2_ or DMOG for 24h. Columns mean of three independent experiments. **B.** Induction of CoCl_2_ increases the expression of endogenous miR-421, while co-transfection of HIF-1α siRNA decreases the expression of miR-421 in SGC-7901 and AGS. Columns mean of three independent experiments. **C.** Induction of CoCl_2_ in HEK293T cells increases the luciferase activity of miR-421 promoter. When 293T cells are co-transfected with Si-HIF-1α, the increased luciferase activity is diminished. Columns mean of three independent experiments. **D.** SGC-7901 xenografted male BALB/c-nude mice (4 weeks old) were treated with BAY 87-2243, a HIF-1α specific inhibitor, for 15 days. QRT-PCR illustrates that BAY 87-2243 reduces the expression of miR-421 by 0.27-fold in gastric cancer tissues. IHC staining confirms that the expression of HIF-1α decreases in BAY 87-2243 treated cancer tissues compared to control group. Columns mean of three independent experiments. ***P < 0.001.

To explore the change of miRNA in gastric cancer cells under hypoxic conditions, a miRNA microchip was used in a previous study [15]. As a result, 43 miRNAs were up regulated by over 2-fold, whereas 12 miRNAs were down regulated by over 2-fold under hypoxic conditions (GSE 56870). Therefore, we used quantitative real-time PCR (qRT-PCR) to confirm the microchip data, and miR-421, miR-130b, miR-129-3p, miR-210, miR-424 and miR-382 were found to be drastically up regulated by treated with CoCl_2_ and DMOG for 24h (Figure 3A). The role of hypoxia-induced miR-421 in cancer progression and drug resistance has not been elucidated in gastric cancer. Therefore, we chose miR-421 as a candidate miRNA in regulation of drug resistance for further study.

Next, we explored the function of HIF-1α in expression of miR-421 under hypoxic conditions. Induction of CoCl_2_ up regulated the expression of endogenous miR-421, while co-transfection of HIF-1α siRNA would down regulate the expression of miR-421 in turn in SGC-7901 and AGS gastric cancer cell lines (Figure 3B). To further investigate regulation of miR-421 by HIF-1α, we used the miRStart database (http://mirstart.mbc.nctu.edu.tw/home.php) to predict the transcription start site (TSS) of miR-421. The result indicated the TSS of miR-421 located 20170 upstream of the precursor. We used JASPAR database (http://jaspar.genereg.net) to predict the conserved HIF-1α binding sequence (Hypoxia Response Element, HRE) in the promoter region of miR-421, and HIF-1α was found to be bound to HRE in the 1 kb upstream of the TSS of miR-421. Therefore, we cloned the promoter region of miR-421 (−1 kb) harboring predicted HRE sequences into pGL3 vector. As a result, treating with CoCl2 in HEK293T cells increased the luciferase activity of miR-421 promoter. When HEK293T cells were co-transfected with Si-HIF-1α, the increased luciferase activity would be diminished (Figure 3C).

Moreover, we treated gastric cancer cell line SGC-7901 xenografted male BALB/c-nude mice (4 weeks old) with BAY 87-2243, a HIF-1α specific inhibitor, for 15 days. We used qRT-PCR to confirm that BAY 87-2243 reduced the expression of miR-421 by 0.27-fold in gastric cancer tissues (Figure 3D). We also used IHC staining to confirm that the expression of HIF-1α decreased in BAY 87-2243 treated cancer tissues compared to control group (Figure 3D).

Epigenetic change also plays an important role in regulation of gene expression. To clarify the potential role of epigenetic regulation of miR-421, SGC-7901 and AGS gastric cancer cell lines were treated with 5-Aza, a methyltransferase inhibitor. We found that the expression of miR-421 was significantly increased in both 5-Aza treated cell lines, suggesting existence of methylation regulation in miR-421 (Supplementary Figure S1A). Next, we used the CpG Island Searcher Program (http://www.urogene.org/methprimer) to identify a CpG island located 6-9 kb upstream of TSS of miR-421, and we conducted sodium bisulfite sequencing assay to assess the methylation status of the predicted CpG island (Supplementary Figure S1B). As a result, the methylation status was drastically increased in SGC-7901 and AGS gastric cancer cell lines. A total of 97.0% and 77.0% of CpGs were methylated in SGC-7901 and AGS cell lines, which further verified the involvement of methylation regulation in miR-421 silencing (Supplementary Figure S1C). Moreover, involvement of CoCl2 increased the expression of DNA methyltransferase 1 and DNA methyltransferase 3a in both cell lines, which could further elevate the methylation level of CpG island of miR-421 and decease the expression of miR-421 (Supplementary Figure S1D). However, as we mentioned before, miR-421 could be significantly increased in gastric cancer cell lines by treated with CoCl2, which was contradicted with the methylation status of miR-421. Therefore, we deduced that HIF-1α was a more powerful factor in miR-421 activation.

To further confirm the regulatory role of HIF-1α in miR-421 activation, flow cytometry and transwell assays were conducted. We assessed the function via. Inhibitor of miR-421 reverses the function of HIF-1α. The results indicated that CoCl2 or miR-421 drastically decrease the percentage of apoptotic cells and increase the migration and invasion ability in cisplatin treated SGC-7901 and AGS cells, which could be reversed by transfection of miRNA-421 inhibitor (miR-421-Inh) together with CoCl2 (Supplementary Figure S2). These results indicated that HIF-1α is a key factor in regulation of miR-421.

### MiR-421 could be a prognostic marker in gastric cancer

**Figure 4:**
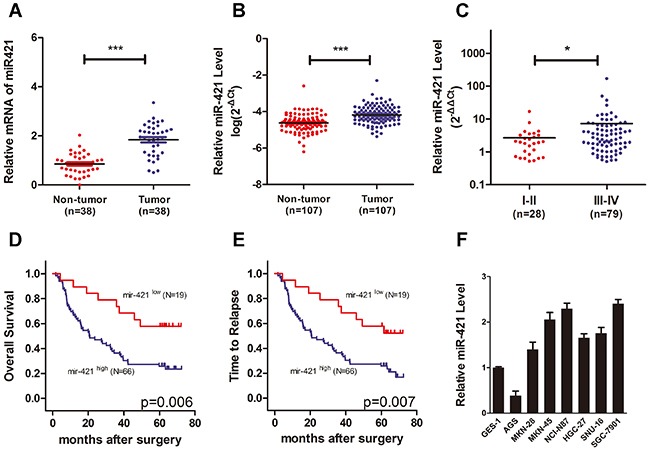
MiR-421 is increased in gastric cancer and associated with poor prognosis **A.** Scatter dot plots show relative mRNA expression of miR-421 is significantly increased in gastric cancer tumor tissues compared with non-tumor tissues using data from The Cancer Genome Atlas. **B.** Scatter dot plots illustrate that the expression of miR-421 is significantly increased in tumor tissues compared with non-tumor ones in a cohort of gastric cancer specimens (n=107). **C.** The expression of miR-421 is higher in advanced gastric cancers compared with localized ones. **D, E.** Kaplan–Meier analysis illustrates that the gastric cancer patients with low levels of miR-421 have a much longer OS and TTR. **F.** QRT-PCR shows relative expression of miR-421 in gastric cancer cell lines (AGS, MKN-28, MKN-45, NCI-N87, HGC-27, SNU-16, and SGC-7901) compared to the immortalized human gastric epithelial cell line GES-1. Columns mean of three independent experiments. OS, overall survival; TTR, time to relapse; *P<0.05; ***P < 0.001.

To investigate the potential role of miR-421 in gastric cancer, the miR-421 expression pattern was evaluated using data from The Cancer Genome Atlas (TCGA). The results showed that the relative mRNA expression of miR-421 was significantly increased in gastric cancer tumor tissues compared with non-tumor tissues in a cohort of 38 gastric cancer patients (Figure 4A). Next, we determined the clinical significance of miR-421 by analyzing its expression pattern in 107 pairs of gastric cancer tissues and para-cancerous tissues via qRT-PCR to further confirm the finding from TCGA (Table 1). As a result, the expression of miR-421 was significantly increased in gastric cancer tissues (p < 0.001) (Figure 4B). In addition, we compared the expression pattern of miR-421 between localized gastric cancer and advanced gastric cancer, and the result indicated that the expression of miR-421 was higher in advanced gastric cancer compared with localized gastric cancer (p < 0.05), indicating miR-421 could be related to cancer aggressiveness and poor prognosis (Figure 4C). Furthermore, Kaplan–Meier analysis illustrated that those patients with low levels of miR-421 had a much longer overall survival (53.8 vs. 32.1 months, p = 0.006) and time to relapse (48.4 vs. 27.0 months, p = 0.007) (Figure 4D, 4E). Cox multivariate analysis illustrated that miR-421 was an independent predictor of overall survival (p = 0.016, HR 2.586, 95%CI 1.194-5.599) and recurrence-free survival (p=0.014, HR 2.465, 95%CI 1.201-5.060) (Table 2). These results indicated that miR-421 was an important prognosis marker in gastric cancer. The expression of miR-421 varied dramatically among gastric cancer cell lines. The immortalized human gastric epithelial cell line GES- 1 showed relatively low expression of miR-421.

**Table 1:** Clinical characteristics of the gastric cancer patients

	Expression of miR-421				
	Low		High		
	No. of patients	%	No. of patients	%	*P* value
**Total**	20		87		
**Age**					0.394
≤ 60	12	60.0	43	49.4	
> 60	8	40.0	44	50.6	
**Gender**					0.563
Male	17	85.0	69	79.3	
Female	3	15.0	18	20.7	
**Tumor differention**					0.627
Undifferentiation	0	0	2	2.3	
Poor	15	75.0	53	60.9	
Moderate	5	15.0	31	35.7	
Well	0	0	1	1.1	
**Tumor stage**					0.150
I–II	8	40.0	21	24.1	
III–IV	12	60.0	66	75.9	
**Tumor site**					0.512
Cardia	3	15.0	25	28.8	
Fundus	1	5.0	1	1.1	
Body	10	50.0	36	41.4	
Antrum	6	30.0	23	26.4	
Pylorus	0	0	2	2.3	
**Vascular invasion**					0.735
Yes	13	65.0	53	60.9	
No	7	35.0	34	39.1	
**Nerve invasion**					0.175
Yes	10	50.0	48	55.2	
No	10	50.0	39	44.8	

**Table 2:** Univariate and multivariate analyses of predictors of overall survival and time to relapse in gastric cancer patients

Variables	Overall Survival							Time to Relapse						
	Death				Univariable analysis	Multivariable analysis		Recurrence				Univariable analysis	Multivariable analysis	
	Yes	No	N	%	*P^a^*	HR (95% CI)	*P^b^*	Yes	No	N	%	*P^a^*	HR (95% CI)	*P^b^*
**Age**														
≤ 60	28	15	43	50.6	0.681			29	14	43	50.6	0.748		
> 60	30	12	42	49.4				32	10	42	49.4			
**Gender**														
Male	48	21	69	81.2	0.489			51	18	69	81.2	0.367		
Female	10	6	16	18.8				10	6	16	18.8			
**Tumor differention**														
Undifferentiated/Poor	40	15	55	64.7	0.083			42	13	55	64.7	0.079		
Moderate/Well	18	12	30	35.3				19	11	30	35.3			
**Vascular invasion**														
Yes	40	13	53	62.4	0.048	0.868	0.667	42	11	53	62.4	0.052		
No	18	14	32	37.6		(0.456-1.655)		19	13	32	37.6			
**Nerveinvasion**														
Yes	42	6	48	56.5	0.000	2.919	**0.002**	43	5	48	56.5	0.000	2.576	**0.001**
No	16	21	37	43.5		(1.464-5.821)		18	19	37	43.5		(1.446-4.590)	
**Tumorstage**														
I-II	5	15	20	23.5	0.000	3.800	**0.006**	7	13	20	23.5	0.000	3.184	**0.006**
III–IV	53	12	65	76.5		(1.474-9.795)		54	11	65	76.5		(1.395-7.268)	
**miR-421**														
High	50	16	66	77.6	0.006	2.586	0.016	52	14	66	77.6	0.007	2.465	0.014
Low	8	11	19	22.4		(1.194-5.599)		9	10	19	22.4		(1.201-5.060)	

HR, hazard risk ratio; 95%CI, 95% confidence interval.

a Statistical analysis was conducted by Kaplan–Meier method (log-rank test).

b Cox proportional hazards regression.

### Overexpression of miR-421 promotes tumor malignant behavior in gastric cancer

**Figure 5:**
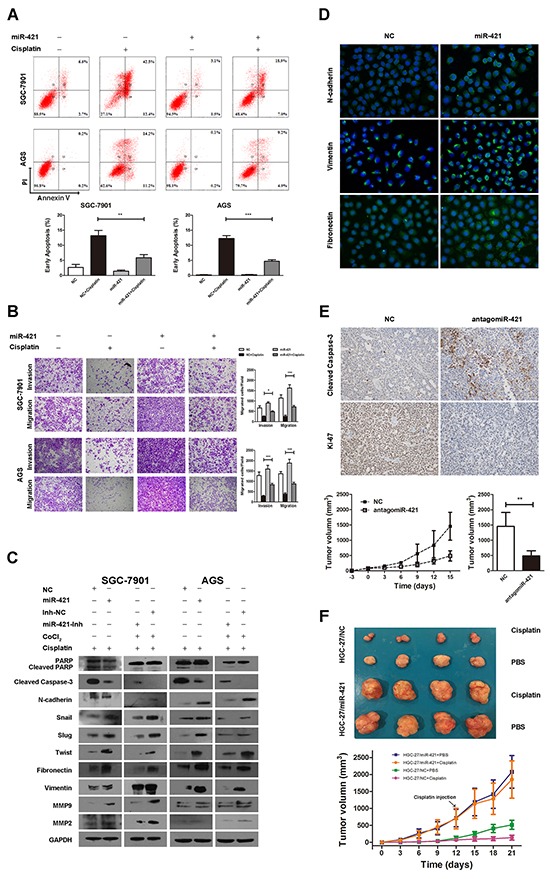
Overexpression of miR-421 promotes metastasis, inhibits apoptosis, and induces cisplatin resistance in gastric cancer **A.** Flow cytometry analysis indicates that cisplatin drastically increases the percentage of early apoptotic apoptotic cells in SGC-7901 and AGS cell lines, which could be attenuated by transfection of miR-421. Representative images and quantitative analysis of cell apoptosis are presented. Columns mean of three independent experiments, and bars SD. **B.** Transwell assays illustrate that cisplatin decreased the migration and invasion ability in both cell lines, and the inhibitory effect could be reversed by transfection of miR-421. Representative migration and invasion images at ×100, and quantitative analysis of transwell assays are presented. Columns mean of three independent experiments, and bars SD. **C.** Western blot analysis shows that the expression of cleaved caspase-3 and cleaved PARP are induced by cisplatin, and N-cadherin, Fibronectin, Vimentin, Snail, Slug, Twist, MMP-9, and MMP-2 decreased, which could be reversed by transfection of miR-421. CoCl_2_ induces low level of cleaved caspase-3 and cleaved PARP, and high level of N-cadherin, Fibronectin, Vimentin, Snail, Slug, Twist, MMP-9, and MMP-2, which could be reversed by transfection of miR-421-Inh. **D.** Immunofluorescence staining illustrates that miR-421 increased the expression of N-cadherin, Fibronectin, and Vimentin compared to NC. **E.** Overexpression of antagomiR-421 retards the growth of SGC-7901 formed tumor in vivo. IHC staining illustrates that tumor derived from antagomiR-421-overexpressed gastric cancer cells shows higher level of cleaved caspase-3 and lower level of Ki-67 compared with the control group. Error bars mean of SD. **F.** Overexpression of miR-421 promotes tumor growth and contributes to cisplatin resistance in vivo. HGC-27/NC and HGC-27/miR-421 cells were subcutaneously implanted. When palpable tumors arose, each group were randomized to be injected intraperitoneally with cisplatin (5mg/kg body weight) or PBS every 3 days. The representative tumors in the 4 groups were illustrated. Tumor volumes in the nude mice of the 4 groups were measured every 3 days. Error bars mean of SD. **P<0.01; ***P < 0.001.

To understand the potential role of miR-421 in regulation of drug resistance in gastric cancer, miR-421 was transfected in cisplatin treated SGC-7901 and AGS gastric cancer cell lines to evaluate its function. To assess whether cisplatin induced apoptosis was affected by miR-421, we assessed the function via flow cytometry. The gastric cell lines treated with NC were used as controls. Flow cytometry analysis indicated that cisplatin drastically increased the percentage of early apoptotic cells in both cell lines. The apoptotic ability was attenuated by transfection of miR-421 (Figure 5A). Besides, western blotting analysis indicated that cisplatin significantly induced the activation of caspase-3 and cleavage of PARP, which could be reversed by transfection of miR-421. On the contrary, CoCl_2_ induced low level of activated caspase-3 and cleavage of PARP could be reversed by transfection of miR-421-Inh (Figure 5C).

To further elucidate the function of miR-421 in gastric cancer, we investigated the effect of miR-421 on cisplatin-treated SGC-7901 and AGS cell motility via transwell assay. The results suggested that cisplatin decreased the migration and invasion ability in both cell lines, and the inhibitory effect could be reversed by transfection of miR-421 (Figure 5B). Besides, we used western blotting to analyze the expression of EMT-related proteins in cisplatin treated cell lines when transfected with miR-421. As a result, the expression of N-cadherin, Fibronectin, Vimentin, Snail, Slug, Twist, MMP-9 and MMP-2 was decreased by cisplatin. However, the decreased expression of N-cadherin, Fibronectin, Vimentin, Snail, Slug, Twist, MMP-9 and MMP-2 were attenuated by transfection of miR-421 in both cell lines (Figure 5C). Additionally, we also found that CoCl_2_ induced high level of N-cadherin, Fibronectin, Vimentin, Snail, Slug, Twist, MMP-9 and MMP-2 could be reversed by transfection of miR-421-Inh (Figure 5C). Furthermore, we also use immunofluorescence staining to evaluate the effect of miR-421 on EMT-related proteins in gastric cancer cell. We found that miR-421 increased the expression of mesenchymal biomarkers N-cadherin, Fibronectin, and Vimentin compared to negative control (NC) (Figure 5D).

To further confirm the function of miR-421 in gastric cancer, the growth rate of SGC-7901 gastric cancer cell with or without antagomiR-421 was calculated after subcutaneous implantation into male BALB/c-nude mice (4 weeks old). As a result, overexpression of antagomiR-421 retarded the growth of tumor *in vivo* (Figure 5E). IHC staining illustrated that tumor derived from antagmiR-421 gastric cancer cells showed higher expression level of activated caspase-3 and lower expression level of Ki-67 compared with the control group (Figure 5E).

In the other hand, cisplatin sensitivity was evaluated *in vivo* with HGC-27 gastric cancer cell, which exhibited moderate basic expression level of miR-421. HGC-27/NC or HGC-27/miR-421 cells were subcutaneous implantated into male BALB/c-nude mice (4 weeks old). Cisplatin (5 mg/kg body weight) or PBS was intraperitoneally injected every 3 days for 4 times. Cisplatin inhibited the growth in both HGC-27/NC and HGC-27/miR-421 groups, and a much more significant reduction of the volume in HGC-27/NC group was observed. The size of PBS treated HGC-27/NC was significantly smaller than that of HGC-27/miR-421, which was consistent with that of SGC-7901 *in vivo*. Furthermore, the growth of HGC-27/miR-421 was faster than that of HGC-27/NC when treated with cisplatin, which suggested that miR-421 contributed to cisplatin resistance. These results indicated that overexpression of miR-421 promoted that tumor malignant behavior of gastric cancer.

### E-cadherin and caspase-3 are targets of miR-421

To identify the potential downstream target of miR-421, TargetScan (http://www.targetscan.org), miRanda (http://www.microrna.org/microrna/hpme.do), and miRWalk (http://www.umm.uni-heidelberg.de/apps/zmf/mirwalk) online databases were used for analysis. Considering the candidate target genes predicted by the three online databases and function of miR-421, E-cadherin and caspase-3 were chose as candidate targets of miR-421. Previous researches reported that E-cadherin and caspase-3 were key regulators in the process of EMT and apoptosis, respectively, and loss function of E-cadherin and caspase-3 were common phenomena in cancer development.

**Figure 6:**
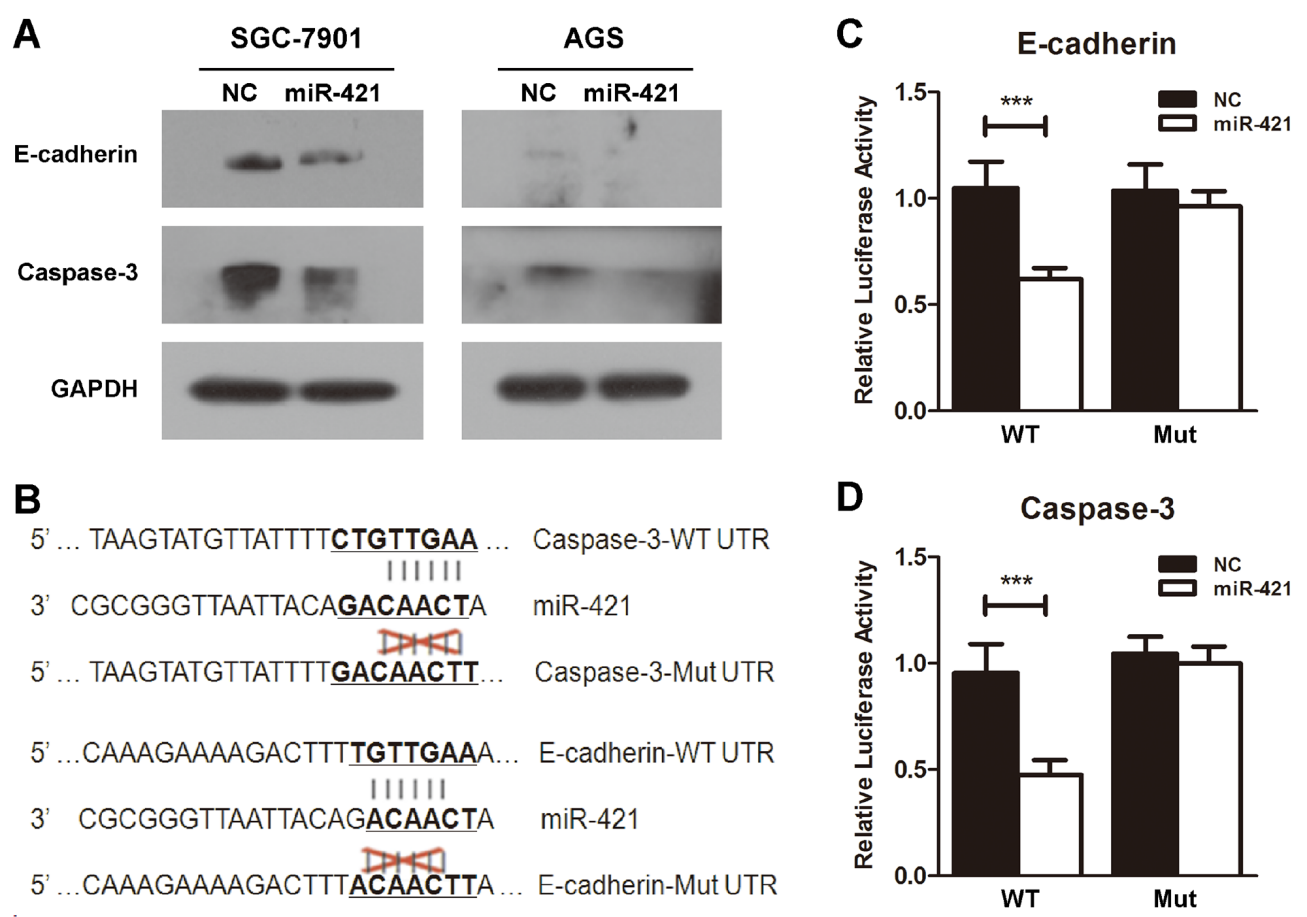
E-cadherin and caspase-3 are targets of miR-421 **A.** Elevated expression of E-cadherin and caspase-3 was determined by western blot when SGC-7901 and AGS cells were transfected with miR-421. **B.** Predicted miR-421 target sequences in the 3′-UTR of E-cadherin and caspase-3 are illustrated. **C.** and **D.** The HEK293T cells transfected with WT-3′-UTR of E-cadherin or caspase-3 vector and miR-421 significantly decrease the relative luciferase activity compared with transfection of WT-3′-UTR vector and NC. Co-transfection with Mut-3′-UTR vector and miR-421 or NC doesn't inhibit the relative luciferase activity. Columns mean of three independent experiments, and bars SD. ***P < 0.001.

To identify whether E-cadherin and caspase-3 were down regulated after the transfection of miR-421, SGC-7901 and AGS cells were transfected with miR-421 for 48h, and the protein level of E-cadherin and caspase-3 were examined. We found that the expression of E-cadherin and caspase-3 were drastically suppressed after transfection of miR-421, indicating E-cadherin and caspase-3 were regulated by miR-421 and could be the downstream targets of miR-421 (Figure 6A).

To further determine whether E-cadherin and caspase-3 were direct downstream targets of miR-421, the 3′-UTR of E-cadherin and caspase-3 were synthesized and cloned into downstream of pmirGLO Dual-Luciferase miRNA Target Expression Vector with firefly luciferase. Besides, vectors with mutated binding sites of E-cadherin and caspase-3 were also constructed (Figure 6B). As a result, the HEK293T cells transfected with WT-3′UTR vector and miR-421 significantly decreased the relative luciferase activity compared with transfection of WT-3′UTR vector and NC. Nevertheless, co-transfection with Mut-3′UTR vector and miR-421 or NC didn't inhibit the relative luciferase activity (Figure 6C, 6D). These results indicated that E-cadherin and caspase-3 were the direct targets of miR-421 based on the luciferase assays.

## DISCUSSION

This study illustrated that miR-421 up regulated by HIF-1α involved in the regulation of drug resistance crosstalk with EMT promotion and apoptosis inhibition. To determine novel drug resistance related miRNAs under hypoxic conditions, we referred to a previous published microRNA microarray (GSE 56870) and used qRT-PCR to verify the data of the microchip. As a result, we chose miR-421 as a candidate miRNA, and used *in vitro* and *in vivo* assays to identify its function in gastric cancer. Furthermore, we also found that miR-421 was an important prognostic marker in gastric cancer. Therefore, targeting miR-421/ E-cadherin/caspase-3 could be a potential strategy for the treatment of cisplatin-resistance gastric cancer patients.

EMT has been identified to be involved in the process of drug resistance in many types of tumor cells [22–27]. Platelet-derived growth factor-D (PDGF-D) pathway was involved in the acquisition of EMT characteristics of gemcitabine resistant hepatocellular carcinoma cells [22]. Induction of EMT contributed to the decreased therapeutic efficacy of oxaliplatin in colorectal cancer, due to the transition of the tumor cells from a proliferative to invasive phenotype [26]. Besides, EMT was also involved in paclitaxel and tamoxifen resistant breast cancer cells [24, 25]. Additionally, blockade of Endothelin-1 (ET-1)/endothelin A receptor driven EMT could overcome cisplatin resistance and improve the outcome of epithelial ovarian cancer patients' treatment [27]. To the best of our knowledge, only a few reports published so far regarding the association between EMT and drug resistance of gastric cancer. Zhang et al [28] illustrated that the aggressive phenotype of adriamycin resistant gastric cancer cells was regulated by induction of EMT and activation of the canonical Wnt/β-catenin pathway, which could be suppressed by pantoprazole which targeted the EMT and Akt/GSK-3β/β-catenin signaling. EMT was also involved in lapatinib resistant HER2-positive gastric cancer [29]. In our study, we found that transfection of HIF-1α-induced-miR-421 significantly down regulated the epithelial biomarker E-cadherin and up regulated N-cadherin, Fibronectin, Vimentin, Snail, Slug, Twist, MMP-2, and MMP-9 in cisplatin treated gastric cancer cell lines. Furthermore, miR-421 drastically increased the migration and invasion ability of cisplatin treated gastric cancer cell lines, indicating that miR-421-regulated EMT was involved in the process of cisplatin resistance in gastric cancer cells.

Inhibitor of miR-944 promoted cisplatin-induced apoptosis through loss of mitochondrial membrane potential and activation of caspase-3 in breast cancer [30]. Elevated HIF-1α induced miR-424 decreased breast cancer sensitivity to doxorubicin by suppressing the expression of PDCD4, known as an apoptosis-related protein, and subsequently inhibiting the expression of caspase-3 and PARP [8]. ARC (Apoptosis Repressor with Caspase Recruitment Domain) is identified as an endogenous inhibitor of apoptosis [31] which has the unusual property of inhibiting both extrinsic and intrinsic pathways [32]. It was reported that elevated expression of endogenous ARC promoted breast carcinogenesis by inducing chemoresistance in invasive cells, and by increasing the proliferation, invasion and metastasis ability of primary tumor [33]. miR-185 was reported to increase gastric cancer sensitivity to cisplatin and doxorubicin by inhibiting the expression of ARC [34]. In our study, we found that HIF-1α-induced miR-421 significantly attenuated the apoptotic ability induced by cisplatin, and reversed the cisplatin induced activation of caspase-3 and cleavage of PARP in gastric cancer cell lines.

Previous studies illustrated that the famous hypoxamiR, miR-210, increased cancer cell proliferation and invasion by targeting various genes [35, 36]. In our study, HIF-1α was stabilized by CoCl_2_ and bound to the HRE site of the promoter region of miR-421, subsequently increasing the expression of miR-421. Besides, transfection of Si-HIF-1α together with induction of CoCl_2_, the increased luciferase activity would be diminished compared to induction of CoCl_2_ alone. Therefore, we identified the HIF-1α induced miR-421 as a hypoxamiR and elucidated its potential function in EMT and apoptosis related drug resistance.

miR-421 has been regarded as an onco-microRNA in neuroblastoma and hepatocellular carcinoma [19, 21]. It was reported that the N-Myc-induced miR-421 down regulated ATM (Ataxia telangiectasia-mutated gene) expression, establishing an N-Myc/miR-421/ATM pathway which contributed to N-Myc-induced carcinogenesis in neuroblastoma [37]. miR-421 induced ATM down regulation was also reported in breast cancer [38]. Moreover, miR-421 has been considered as an anti-apoptosis miRNA in nasopharyngeal carcinoma and gastric cancer [20, 39]. In our study, we observed that overexpression of miR-421 promoted tumor behavior in gastric cancer and the expression of miR-421 was significantly increased in gastric cancer tissues compared with para-cancerous tissues (p < 0.01) as previously reported [40]. Furthermore, for the first time, we reported that the expression of miR-421 was higher in advanced gastric cancer compared with localized gastric cancer (p < 0.01). Besides, those patients with low levels of miR-421 had a much longer overall survival (p = 0.006) and recurrence-free survival (p = 0.007), and miR-421 was also an independent predictor of overall survival (p = 0.016, HR 2.586, 95%CI 1.194-5.599) and recurrence-free survival (p=0.014, HR 2.465, 95%CI 1.201-5.060) by Cox multivariate analysis, indicating miR-421 could be related to cancer aggressiveness and poor prognosis.

In conclusion, our study illustrated miR-421, a HIF-1α induced miRNA, played an important role in gastric cancer. Overexpression of miR-421 promoted metastasis, inhibited apoptosis, and induced cisplatin resistance in gastric cancer by targeting E-cadherin and caspase-3. Besides, the expression of miR-421 was detected to be higher in advanced gastric cancers compared with localized ones, and significantly increased in tumor tissues compared with non-tumor ones. Therefore, miR-421 could serve as an important prognostic marker and a potential molecular target for therapy in gastric cancer.

## MATERIALS AND METHODS

### Cell lines and cell culture

AGS, SGC-7901, NCI-N87 and HGC-27 human gastric cancer cell lines, and HEK293T cell line were obtained from the Cell Bank of Type Culture Collection of Chinese Academy of Sciences (Shanghai, China). MKN-28, MKN-45, SNU-16 gastric cancer cell lines, and the immortalized human gastric epithelial cell line GES-1 were obtained from 3DBiopharm Biotech Co. Ltd. All cell lines were verified by short tandem repeat (STR) DNA profiling analysis. Cells were cultured in F12K (AGS) (Gibco), RPMI1640 medium (SGC-7901) (Gibco) containing 10% heat-inactivated fetal bovine serum (Gibco), 50U/mL penicillin and 50μg/mL streptomycin in a humid atmosphere at 37°C with 5% CO_2_.

### RNA isolation and real-time PCR

Expression level of miR-421 in cell lines was calculated by quantitative real-time PCR (qRT-PCR). Small RNA was extracted from cell lines with RNAiso Kit for Small RNA (TaKaRa, Japan) and subsequently reverse transcribed into cDNA with One Step PrimeScript miRNA cDNA Synthesis Kit (TaKaRa, Japan). Total RNA was extracted from cell lines with RNAiso plus (TaKaRa, Japan) and transcribed into cDNA using PrimeScript RT reagent Kit (TaKaRa, Japan). cDNAs were quantified by SYBR Premix Ex Taq (TaKaRa, Japan) by ABI 7500 fast real-time PCR System (Applied Biosystems, Carlsbad, USA). Small nuclear RNA U6 and GAPDH mRNA were used as internal controls for normalization. All primers were listed in Supplementary Table S1.

### Western blotting analysis

Western blotting analysis was conducted as previously described [41]. The following primary immunoblotting antibodies were used: anti-GAPDH, anti-E-cadherin, anti-Fibronectin, anti-Vimentin, anti-Snail, anti-Slug, anti-Twist, anti-MMP-9, anti-MMP-2, anti-pro caspase-3, and anti-active caspase-3 (Epitomics, Burlingame, USA), anti-HIF-1α and anti-β-catenin (Proteintech, Chicago, USA), anti-N-cadherin (Cell Signaling Technology, Beverly, MA), and poly (ADP-ribose) polymerase (PARP) (Santa Cruz, CA).

### Immunohistochemistry (IHC) staining

IHC staining was performed as previously described [42]. In short, tumor tissues from mice were dewaxed and rehydrated before conducting antigen retrieval. Slides were incubated with anti-HIF-1α (Cell Signaling Technology, Beverly, MA), anti-ki-67 or anti-active caspase-3 (Epitomics, Burlingame, USA) at 4°C overnight, followed by incubation with an HRP-conjugated secondary antibody at room temperature for 1 hour (h). Diaminobenzidine (DAB) was used for coloration, and dark brown was considered to be positive. The strength of positivity was quantified by considering the percentage of positive cells and the staining intensity.

### Immunofluorescence analysis

Gastric cancer cells grown on cover slip were fixed in 4% paraformaldehyde (Invitrogen, Cergy-Pontoise, France) for 15 minutes (min). After washed three times with PBS, unspecific sites were blocked using PBS containing 5% BSA at room temperature for 1 h. Cells grown on cover slip were then incubated with anti-N-cadherin (Cell Signaling Technology, Beverly, MA), anti-Fibronectin and anti-Vimentin (Epitomics, Burlingame, USA) at 4°C overnight, and incubated with the secondary antibodies Alexa Fluor (Molecular Probes, Life Technologies, Saint-Aubin, France) in the dark at room temperature for 1 h. DAPI were used for Nuclei staining. All stained cells were examined and photographed with a Leica SP5 confocal fluorescence microscope.

### Transfection of miRNA mimics, miRNA inhibitor, antagomiRNA, small interfering RNA, and small activating RNA

miRNA-421 (miR-421), miRNA-421 inhibitor (miR-421-Inh), antagomiRNA-421 (antagomiR-421), small interfering RNA-E-cadherin (si-E-cadherin), small interfering RNA- HIF-1α (si-HIF-1α), small activating RNA-E-cadherin (ds-E-cadherin) [43] and negative control (NC) were purchased from GenePharma (Shanghai, China). Transfection was conducted via Lipofectamine 2000 (Invitrogen, Carlsbad, CA, USA) according to the manufacturer's instructions. All the small RNAs were used at a final concentration of 50nM.

### Lentivirus infection

The miR-421-overexpressing lentivirus was supplied by Hanyin (Shanghai, China). HGC-27 cells were infected with the miR-421-overexpressing lentivirus (HGC-27/miR-421) or corresponding mock lentivirus (HGC-27/NC) as previously described [44]. Briefly, virus was added into the cells with 8μg/mL Polybrene® (Sigma-Aldrich, St Louis, MO, USA) for 6-8h, and then fresh culture medium was added. Forty-eight hours later, the infected cells were subjucted to selection with 2μg/mL puromycin

### Cell viability assay

Gastric cancer cells (4000 cells/well) were seeded into 96-well plate. After 24h incubation, the cells were treated with cisplatin (6.25-200μM) and CoCl_2_ (100μM) or cisplatin alone for 48h incubation. Cell Counting Kit-8 (CCK-8, Dojindo, Kumamoto, Japan) was then added to each well after medium in it was removed and incubated for another 2h. Cell viability was measured at a solution absorbance of 450nm with MRX II absorbance reader (Dynex Technologies, Chantilly, VA, USA).

### Colony formation assay

AGS and SGC-7901 gastric cancer cells were incubated for 24h after treated with cisplatin (IC50) and CoCl_2_/ 2′-O-Methyl modified miR-421 or cisplatin alone. Five hundreds of treated cells were then seeded into a new six-well plate and cultivated for another 14 days. Cells were subsequently fixed and stained with absolute methanol and 0.1% crystal violet. The colony formation rate was calculated using the following equation: colony formation rate = (number of colonies/number of seeded cells) ×100%.

### Cell apoptosis assay

Apoptosis was assessed by annexin V-FITC (Invitrogen, Carlsbad, CA, USA) and flow cytometry. Cells were incubated for 24h after treated with cisplatin (IC50) and CoCl_2_/miR-421 or cisplatin alone at a density of 1 × 10^6^ cells in a 6-well plate. The cells were then washed twice with pre-chilled PBS, and resuspended in 1× binding buffer (1 × 10^6^ cells/mL). Cells (100μL) were stained with 5μL annexin V-FITC and 5μL propidium iodide (PI) for 15 min, and 400μL 1× binding buffer was added. Analysis was conducted using a FC500 flow cytometer with CXP software (Beckman Coulter, Fullerton, CA, USA) within 1 h.

### Transwell assay

The 24-well Boyden chamber with 8μm pore size polycarbonate membrane (Corning, NY) was used for evaluating the cell migration. We used Matrigel (BD, Franklin Lakes, NJ) to pre-coat the membrane to simulate a matrix barrier for evaluating the cell invasion. Forty thousand cells were seeded in the upper chamber with 150μL serum-free medium after treated with cisplatin (IC50) and CoCl_2_/miR-421 or cisplatin alone for 24h. As a chemoattractant, a total of 600μL medium with 10% serum was added to each lower chamber. After 24h incubation, the membranes were washed with PBS, fixed with methanol and stained with 0.1% crystal violet. We randomly selected 5 visual fields (×100) from each membrane, and the number of cells that had migrated was counted using an IX71 inverted microscope (Olympus Corp, Tokyo, Japan).

### Luciferase reporter assays

The putative promoter region of miR-421 (−1 kb) harboring predicted hypoxia response element (HRE) sequences were synthesized by PCR (PrimerSTAR, TaKaRa, Japan) and cloned into pGL3 vector between SacI and NheI. CoCl_2_ or DMOG treated HEK293T cells were co-transfected with cloned pGL3 vector and renilla luciferase vector with or withour Si-HIF-1α. After 48h transfection, the relative luciferase activity was calculated by Dual-Luciferase Reporter Assay System (Promega, USA).

The 3′-UTR (untranslated region) of E-cadherin and caspase-3, containing putative target region of miR-421, was synthesized (Sangon, Shanghai, China) and cloned into sites between SacI and SalI downstream of luciferase reporter gene of pmirGLO Dual-Luciferase miRNA Target Expression Vector (Promega, USA), respectively. Additionally, the mutant miR-421 putative target region was inserted into pmirGLO Dual-Luciferase Vector in the same way. All insertions were verified by sequencing (Sangon, Shanghai, China). HEK293T cells were seeded in a 24-well plate for 24h before co-transfected with 50nM of either miR-421 or NC and 200ng reporter plasmid containing wild type (WT) or mutant type (Mut) of E-cadherin or caspase-3 3′-UTR. After 48h transfection, the relative luciferase activity was calculated by Dual-Luciferase Reporter Assay System (Promega, USA).

### 5-Aza-2′-deoxycytidine treatment of the AGS and SGC-7901 gastric cancer cells

AGS and SGC-7901 gastric cancer cells were treated with 10μL 5-Aza-2′-deoxycytidine (5-Aza) (5μM dissolved in DMSO) (Sigma A3656) for 4 days. RNA was extracted and analyzed for the expression of miR-421.

### DNA methylation analysis

Genomic DNA from AGS and SGC-7901 gastric cancer cell lines was bisulfite modified and the CpG islands were amplified by PCR (Primers were listed in Supplementary Table S1). The PCR products were separated by agarose gel electrophoresis (3%), extracted and cloned into the pUC18 T-vector (Sangon, China). After bacterial amplification of the cloned PCR fragments by standard procedures, 10 clones were sent for DNA sequencing (Sangon, China).

### Xenograft experiments

Male BALB/c-nude mice (4 weeks old) were purchased from the Shanghai Experimental Animal Center, Chinese Academy of Sciences (Shanghai, China). SGC-7901 gastric cancer cells (1 × 10^7^ in 200μL PBS) were injected subcutaneously into each mouse. When palpable tumors arose, the mice in group 1 were treated with BAY 87-2243 (0.5mg/kg body weight) [45], a highly selective inhibitor of HIF-1α, every 3 days for 15 days. The mice in group 2 were injected intratumorally with 30μg of Lipofectamine 2000-encapsulated antagomiR-421 or NC every 3 days for 15 days.

In the cisplatin sensitivity assays, male BALB/c-nude mice (4 weeks old) were injected subcutaneously with either HGC-27/NC or HGC-27/miR-421 cells (1 × 10^7^ in 200μL PBS) [46]. Each group were randomized when palpable tumors arose, and respectively treated with intraperitoneal injection of cisplatin (5 mg/kg body weight) or PBS every 3 days [47, 48].

Tumor size was measured by 2 perpendicular diameters via caliper every 3 days. Tumor volume was calculated with the following formula V = (width^2^ × length × 0.52).

### Patients and samples

A total of 107 specimens of primary gastric adenocarcioma specimens were obtained by surgical resection between 2007 and 2010 at Fudan University Shanghai Cancer Center (Shanghai, China). All the 107 matched fresh frozen gastric adenocarcioma tissues (C) and adjacent non-cancerous tissues (N) were selected for qRT-PCR. In order to determine the prognostic factor, follow-up was conducted, and the outcome of 85 patients was determined. Tumor staging was determined according to the tumor–node–metastasis classification system of the American Joint Committee on Cancer, 7th edition. Follow-up was performed every 6 months by specially trained staff according to standard epidemiologic procedures. Patients were acquired with informed consent, under the protocol approved by Fudan University Shanghai Cancer Center research ethics committee.

### Statistical analysis

All the statistics were expressed as mean ± standard deviation (SD) of three independent experiments. The student's t-test or One-way ANOVA was used to evaluate the between-group difference. Overall survival and recurrence-free survival were analyzed by the Kaplan–Meier method, and the log-rank test was used to estimate the differences between groups. Cox's proportional hazard model was used to assess independent prognostic indicators in the multivariate analysis. SPSS for Windows v.16.0 (SPSS, Chicago, IL) and GraphPad Prism 5.0 (GraphPad Software, La Jolla, CA) were used to conduct all the relative analyses. P < 0.05 was set to be statistically significant.

## SUPPLEMENTARY FIGURES AND TABLE


