# Supplementary material for: MicroRNA-421 regulated by HIF-1α promotes metastasis, inhibits apoptosis, and induces cisplatin resistance by targeting E-cadherin and caspase-3 in gastric cancer
Source: Oncotarget. 2016 Mar 21;7(17):24466–82. doi: 10.18632/oncotarget.8228 (PMC5029715; doi:10.18632/oncotarget.8228)
Supplement: Supplementary file 1 [file oncotarget-07-24466-s001.pdf]

SUPPLEMENTARY FIGURES AND TABLE

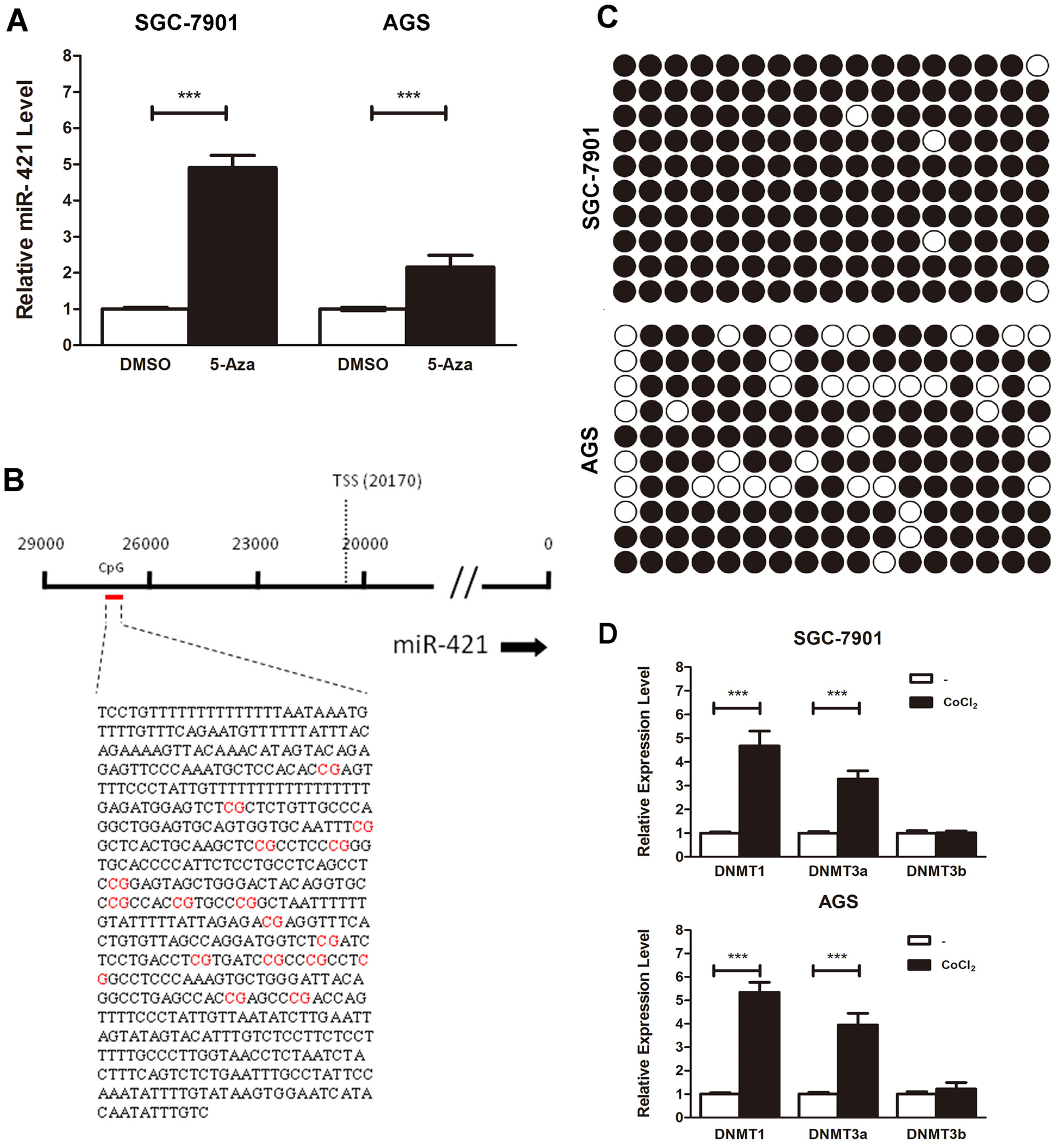

Supplementary Figure S1: DNA methylation is involved in miR-421 regulation. A. DNA methylation inhibitor 5-Aza increases the expression of miR-421 in SGC-7901 and AGS gastric cancer cell lines. **B.** Region of CpG Island of miR-421 was predicted by the CpG Island Searcher Program. **C.** The promoter of miR-421 is hypermethylated in gastric cancer cell lines SGC-7901 and AGS. The filled and open circles represent the methylated and unmethylated CpGs from ten independent randomly chosen colonies. **D.** Involvement of $CoCl_2$ increases the expression of DNMT1 and DNMT 3a in both cell lines (RNA level). Columns mean of three independent experiments, and bars SD. ***$P < 0.001$.

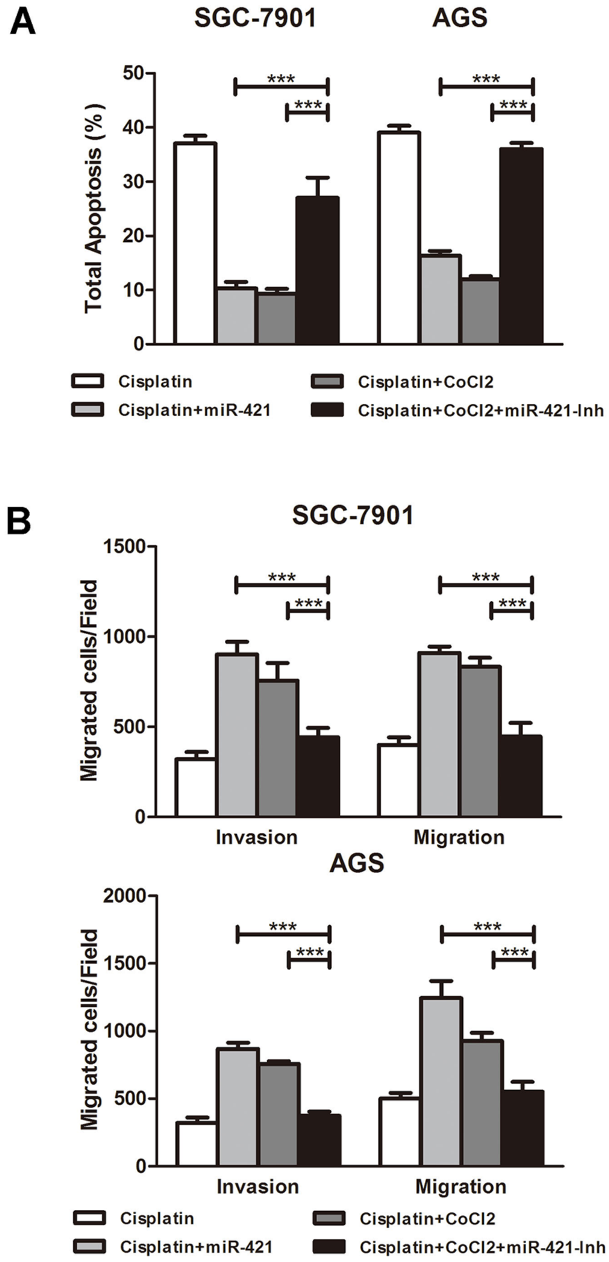

Supplementary Figure S2: MiR-421-Inh reverses the function of HIF-1α. A. Flow cytometry analysis indicates that $CoCl_2$ or miR-421 drastically decreases the percentage of apoptotic cells in cisplatin treated SGC-7901 and AGS, and transfection of miR-421-Inh together with $CoCl_2$ functions inversely (histograms are presented). **B.** Transwell assays illustrate that $CoCl_2$or miR-421 drastically increases the migration and invasion ability in cisplatin treated SGC-7901 and AGS, and transfection of miR-421-Inh together with $CoCl_2$ functions inversely (quantitative analysis is presented). Columns mean of three independent experiments, and bars SD. ***$P < 0.001$.

Supplementary Table S1: The oligonucleotides used in this study

Name[a]	Sequence (5′->3′)
miR-421 mimics (sense)	AUCAACAGACAUUAAUUGGGCGC
miR-421-Inh (sense)	GCGCCCAAUUAAUGUCUGUUGAU
antagomiR-421 (sense)	GCGCCCAAUUAAUGUCUGUUGAU
NC (sense)	ACUACUGAGUGACAGUAGA
NC-Inh (sense)	UCUACUCUUUCUAGGAGGUUGUGA
Si-HIF-1α (sense)	UCAAGUUGCUGGUCAUCAG
Si-E-cadherin (sense)	GCCACAUACACUCUCUUCUTT GAGGCUGUAUACACCAUAUTT GGGACAACGUUUAUUACUATT
dsE-cadherin (sense)	AACCGUGCAGGUCCCAUAATT
miR-421 F	ATCAACAGACATTAATTGGGCGC
miR-210 F	AGCCCCTGCCCACCGCACACTG
miR-424 F	CAAAACGTGAGGCGCTGCTAT
miR-130b F	ACTCTTTCCCTGTTGCACTAC
miR-129-3p F	AAGCCCTTACCCCAAAAAGTAT
miR-382 F	GAAGTTGTTCGTGGTGGATTCG
U6 F	TGCGGGTGCTCGCTTCGGCAGC
DNMT 1 F	AGGTGGAGAGTTATGACGAGGC
DNMT 1 R	GGTAGAATGCCTGATGGTCTGC
DNMT 3a F	CCTCTTCGTTGGAGGAATGTGC
DNMT 3a R	GTTTCCGCACATGAGCACCTCA
DNMT 3b F	TAACAACGGCAAAGACCGAGGG
DNMT 3b R	TCCTGCCACAAGACAAACAGCC
miR-421 promoter F	tcgaGCTAGCCACCACCACTCCTGGCTAA
miR-421 promoter R	tcgaAAGCTTCAATCCTCCCTCCTCACC
CpG F	TTTTAGAATGTTTTTTATTTATAGAAAAG
CpG R	CAAAATATTAACAATAAAAAAAAACTAATC
GAPDH F	AAGGTGAAGGTCGGAGTCA
GAPDH R	GGAAGATGGTGATGGGATTT
E-cadherin-Wt F	cACAATCAAAGAAAAGACTTTTGTTGAAATAGCTTTACTGTTTCTCAAg
E-cadherin -Wt R	tcgacTTGAGAAACAGTAAAGCTATTTCAACAAAAGTCTTTTCTTTGATTGTgagct
E-cadherin -Mut F	cACAATCAAAGAAAAGACTTTacaacttATAGCTTTACTGTTTCTCAAg
E-cadherin -Mut R	tcgacTTGAGAAACAGTAAAGCTATaagttgtAAAGTCTTTTCTTTGATTGTgagct
Caspase-3-Wt F	cATTCTTAAGTATGTTATTTTCTGTTGAAGTTTACAATCAAAGGAAAATg
Caspase-3-Wt R	tcgacATTTTCCTTTGATTGTAAACTTCAACAGAAAATAACATACTTAAGAATgagct
Caspase-3-Mut F	cATTCTTAAGTATGTTATTTTgacaacttGTTTACAATCAAAGGAAAATg
Caspase-3-Mut R	tcgacATTTTCCTTTGATTGTAAACaagttgtcAAAATAACATACTTAAGAATgagct

[a] F, forward primer; R, reverse primer.
